# Covering Large-Dimensional Euclidean Spaces by Random Translates of a Given Convex Body

**DOI:** 10.1007/s00454-026-00871-5

**Published:** 2026-10-29

**Authors:** Boris Bukh, Jun Gao, Xizhi Liu, Oleg Pikhurko, Shumin Sun

**Affiliations:** 1 Department of Mathematical Sciences, Carnegie Mellon University, Pittsburgh, USA; 2 Academy of Mathematics and Systems Science, Chinese Academy of Sciences, Beijing, 100084, China; 3 School of Mathematical Sciences, University of Science and Technology of China, Hefei, China; 4 Mathematics Institute and DIMAP, University of Warwick, Coventry, UK

**Keywords:** Covering density, Covering multiplicity, Sphere coverings, Convex bodies, Probabilistic method, Primary 52C17, Secondary 05B40, 05D40

## Abstract

Determining the minimum density of a covering of $\mathbb {R}^{n}$ by Euclidean unit balls as $n\rightarrow \infty$ is a major open problem, with the best known results being the lower bound of $\left( \textrm{e}^{-3/2}+o(1)\right) n$ by Coxeter, Few and Rogers [*Mathematika* **6**, 1959] and the upper bound of $\left( 1/2+o(1) \right) n \ln n$ by Dumer [*Discrete Comput. Geom.* **38**, 2007]. We prove that there are ball coverings of $\mathbb {R}^n$ attaining the asymptotically best known density $\left( 1/2+ o(1)\right) n \ln n$ such that, additionally, every point of $\mathbb {R}^n$ is covered at most $\left( 1.79556... + o(1)\right) n \ln n$ times. This strengthens the result of Erdős and Rogers [*Acta Arith.* **7**, 1961/62] who had the maximum multiplicity at most $\left( \textrm{e} + o(1)\right) n \ln n$. On the other hand, we show that the method that was used for the best known ball coverings (when one takes a random subset of centres in a fundamental domain of a suitable lattice in $\mathbb {R}^n$ and extends this periodically) fails to work if the density is less than (1/2+o(1))nlnn; in fact, this result remains true if we replace the ball by any convex body *K*. Also, we observe that a “worst” convex body *K* here is a cube, for which the covering density coming from random constructions is only (1+o(1))nlnn.

## Introduction

The fundamental result of Rogers [15] states that, for every *convex body* *K* (i.e. a compact convex set with non-empty interior) in the *n*-dimensional Euclidean space $\mathbb {R}^n$, there exists a covering of $\mathbb {R}^{n}$ by translates of *K* with density at most (1+o(1))nlnn as $n\rightarrow \infty$. If *K* is a ball then the covering density was improved to (1/2+o(1))nlnn by Dumer [8] while the best known lower bound is $\left( \textrm{e}^{-3/2} + o(1)\right) n$ by Coxeter, Few and Rogers [6]. Closing this gap for ball covering is a major open problem.

In brief, the new results proved in this paper are as follows. First, Theorem 1.1 proves that there are ball coverings of $\mathbb {R}^n$ attaining the asymptotically best known density $\left( 1/2+o(1) \right) n \ln n$ such that, additionally, every point of $\mathbb {R}^n$ is covered at most $\left( 1.79556... + o(1)\right) n \ln n$ times. This strengthens a result of Erdős and Rogers [9] who had the maximum multiplicity at most $\left( \textrm{e} + o(1)\right) n \ln n$. Also, Theorem 1.3 shows that, for any given convex body $K\subseteq \mathbb {R}^n$, the method that was used in [8, 15] (when one takes a random subset of centres in a fundamental domain of a suitable lattice in $\mathbb {R}^n$ and extends this periodically) fails with high probability to produce a *K*-covering if the appropriately defined density is strictly less than (1/2+o(1))nlnn. Finally, Theorem 1.5 gives that, somewhat surprisingly, a “worst” convex body *K* for this method is a cube, for which the covering density coming from random constructions is only (1+o(1))nlnn (which matches the bound of Rogers [15]).

Let us provide the details. Let

$$ B_r^n(\textbf{x}):=\{\textbf{y}\in \mathbb {R}^{n}:\Vert \textbf{x}-\textbf{y}\Vert \le r\} $$

denote the closed ball of radius *r* centred at $\textbf{x}$, where $\Vert \cdot \Vert$ denotes the Euclidean norm. When $\textbf{x} = \textbf{0}$ is the origin of $\mathbb {R}^n$, we write $B_{r}^{n}$ for brevity. Let $\operatorname {vol}(\cdot )$ denote the Lebesgue measure on $\mathbb {R}^n$. Also, we define $\nu _{n}:=\operatorname {vol}(B_1^n)$ to be the volume of the *n*-dimensional unit ball $B_{1}^{n}$.

Let $K \subseteq \mathbb {R}^{n}$ be a convex body. A (discrete) set $P \subseteq \mathbb {R}^{n}$ is called a *K-covering* of a set $A \subseteq \mathbb {R}^{n}$ if *A* is a subset of

$$\begin{aligned} P + K :=\left\{ \textbf{p} + \textbf{x} :\textbf{p} \in P \text { and } \textbf{x} \in K \right\} , \end{aligned}$$

that is, the union of the translates of *K* by the elements of *P* contains every point of *A*. For a measurable $A\subseteq \mathbb {R}^n$ of finite positive measure, the *covering density* of a set $P\subseteq \mathbb {R}^n$ over *A* is defined as

$$\begin{aligned} \vartheta _{K}(P, A) :=\sum _{\textbf{p} \in P} \frac{\operatorname {vol}\big ( A\cap (K+\textbf{p}) \big )}{\operatorname {vol}(A)}. \end{aligned}$$

The *covering multiplicity of a point* $\textbf{x} \in A$ is defined as

$$\begin{aligned} \mu _{K}(P, \textbf{x}) :=\left| \left\{ \textbf{p} \in P :\textbf{x} \in K + \textbf{p} \right\} \right| . \end{aligned}$$

The *covering multiplicity of* *P* is then defined as

$$\begin{aligned} \mu _{K}(P, A) :=\sup \left\{ \mu _{K}(P, \textbf{x}) :\textbf{x} \in A\right\} , \end{aligned}$$

i.e., the maximum number of times that a point of *A* is covered by the translates of *K* by the elements of *P*. The *K-covering density* and *K-covering multiplicity* of *P* over $\mathbb {R}^{n}$ are defined by

$$\begin{aligned} \vartheta _K(P) :=\limsup _{r\rightarrow \infty } \vartheta _K\big (P, [-r,r]^n\big ) \quad \text {and}\quad \mu _K(P) :=\mathop {\textrm{sup}}\limits _{r>0} \mu _K\big (P, [-r,r]^n\big ). \end{aligned}$$

In the case when *K* is the unit ball $B_1^n$, we may omit the subscript *K* and write just ϑ(P,A), $\mu (P, \textbf{x})$, μ(P,A), etc. Thus, for example, Dumer’s result [8] gives a unit ball covering *P* of $\mathbb {R}^n$ with $\vartheta (P)\le \left( 1/2+o(1)\right) n \ln n$. Our first result shows that, additionally, one can assume that the covering multiplicity of *P* is at most (1.795...+o(1))nlnn, improving on the previous multiplicity bound $(\textrm{e}+o(1))n\ln n$ of Erdős and Rogers [9], where $\textrm{e}=2.718...$ is the base of the natural logarithm.

### Theorem 1.1

There exists a unit ball covering *P* of $\mathbb {R}^{n}$ such that

$$\begin{aligned} \vartheta (P) \le \left( \frac{1}{2} + o(1)\right) n \ln n \quad \text {and}\quad \mu (P) \le \big ( \xi + o(1) \big ) n \ln n, \end{aligned}$$

where ξ=1.79556... is the unique (positive) real root of

1 $$\begin{aligned} \ln \left( \frac{2x}{\textrm{e}}\right) = \frac{1}{2x}. \end{aligned}$$

Recall that a *lattice* $\Lambda \subseteq \mathbb {R}^{n}$ is a set of the form

$$\begin{aligned} \Lambda = \left\{ \lambda _1 \textbf{b}_{1} + \cdots + \lambda _n \textbf{b}_{n} :\lambda _i \in \mathbb {Z} \text { for } i \in [n] \right\} , \end{aligned}$$

for some linearly independent vectors $\textbf{b}_{1}, \ldots , \textbf{b}_{n} \in \mathbb {R}^{n}$, where we use the shorthand $[n] :=\{1,\dots ,n\}$. For a convex body $K\subseteq \mathbb {R}^n$, we call a lattice $\Lambda \subseteq \mathbb {R}^{n}$ a *packing lattice* of *K* if, for every two distinct elements $\textbf{p},\textbf{p}'\in \Lambda$, the translates $K+\textbf{p}$ and $K+\textbf{p}'$ are disjoint. The corresponding quotient $\mathbb {T}_{\Lambda }:=\mathbb {R}^n/\Lambda$ (of the topological Abelian group $(\mathbb {R}^n,+)$ by the discrete subgroup Λ) is then called a *packing torus* of *K*. Slightly abusing notation, we use $\operatorname {vol}(\cdot )$ also to denote the measure on $\mathbb {T}_{\Lambda }$ obtained by identifying the torus with a fundamental domain of Λ and using the Lebesgue measure on the latter. Likewise, we may use the same symbol *A* to refer to the projection of $A\subseteq \mathbb {R}^n$ to $\mathbb {T}$, etc.

Recall that a discrete random variable $\textbf{X}$ has the *Poisson distribution with mean* $\lambda \ge 0$, denoted $\textbf{X} \sim \textrm{Pois}(\lambda )$, if for each integer k≥0 the probability of $\textbf{X}$ assuming value *k* is ${\lambda ^{k} \textrm{e}^{-\lambda }}{/k!}$.

A random set $\textbf{X} \subseteq A$ is said to be sampled from the *Poisson point process* on a measurable subset $A\subseteq \mathbb {R}^n$ with intensity ρ if the following conditions hold:

(i) For every bounded measurable set S⊆A, the number of points in *S*, that is, $|\textbf{X} \cap S|$, has the Poisson distribution with mean $\rho \cdot \operatorname {vol}(S)$.
(ii) For any disjoint bounded measurable sets $S_1,\dots ,S_k\subseteq A$, the random variables $|\textbf{X} \cap S_1|,\dots ,|\textbf{X} \cap S_k|$ are mutually independent.

Such a distribution is known to exist and to be uniquely determined by the above two properties.

As in many upper-bound constructions, the covering set $P \subseteq \mathbb {R}^{n}$ that we construct will be of the form Λ+X, where $\Lambda \subseteq \mathbb {R}^{n}$ is a suitable lattice and *X* is a typical (under some distribution) finite subset of the corresponding torus $\mathbb {T}_{\Lambda } :=\mathbb {R}^{n}/\Lambda$. Specifically, Theorem 1.1 will follow from the following result.

### Theorem 1.2

Let 0<δ<1/2 be a real number and M>0 be a constant. Suppose that $\mathbb {T}$ is a packing torus of $B_{2}^{n}$ with $\operatorname {vol}(\mathbb {T}) \le M^n \nu _{n}$. Suppose that the set $\textbf{X}$ is sampled from the Poisson point process on $\mathbb {T}$ with intensity

2 $$\begin{aligned} \rho :=\left( \frac{1}{2} + \delta \right) \frac{n \ln n}{\nu _{n}}. \end{aligned}$$

Then the probability that all the following events occur tends to 1 as $n \rightarrow \infty$:

(i) $$\mathbb {T} \subseteq \textbf{X} + B_{1}^{n}$$,
(ii) $$\vartheta (\textbf{X}, \mathbb {T}) \le \left( \frac{1}{2} + 2\delta \right) n \ln n$$, and
(iii) $\mu (\textbf{X},\mathbb {T}) \le \left( \xi + 10 \delta \right) n \ln n$, where ξ=1.79556... is the unique real root of (1).

The derivation of Theorem 1.1 from Theorem 1.2 is straightforward, given the known results on the existence of reasonably good packing lattices for balls, for which e.g. the classical Minkowski–Hlawka theorem suffices (and we refer the reader to Klartag [11] and Abuya et al. [1] for the best known bounds in high dimensions). Also, note the result of Rogers [14] that, for any convex body $K\subseteq \mathbb {R}^n$, there exists a packing lattice Λ of *K* such that $\operatorname {vol}\big (\mathbb {T}_{\Lambda }\big ) \le 4^n\cdot \operatorname {vol}(K)$.

Our next result shows that the constant 1/2 in (2) cannot be improved using this approach, in fact not only for a ball but for any convex body.

### Theorem 1.3

Let δ>0 be a real number. Let $K \subseteq \mathbb {R}^{n}$ be a convex body and $\mathbb {T}$ be a packing torus of $K-K:=\{\textbf{x}-\textbf{y}:\textbf{x},\textbf{y}\in K\}$. Suppose that $\textbf{X} \subseteq \mathbb {T}$ is sampled from the Poisson point process with intensity

$$\begin{aligned} \rho :=\frac{1}{\operatorname {vol}(K)}\left( \frac{n \ln n}{2} - (1+ \delta ) n \ln \ln n \right) . \end{aligned}$$

Then the probability that $\mathbb {T} \subseteq \textbf{X} + K$ tends to 0 as $n\rightarrow \infty$.

Note that, by Theorem 1.2, the constant 1/2 in front of nlnn in Theorem 1.3 is best possible when *K* is a unit ball and $\operatorname {vol}(\mathbb {T}) \le M^n \nu _{n}$, where *M* is some constant.

### Remark 1.4

If we condition a Poisson point process on resulting in exactly *N* points then we get *N* independent uniform points in the domain. Thus, by monotonicity and concentration (cf Claim 2.3), the conclusion of Theorem 1.3 also holds if $\textbf{X}$ is obtained by choosing *N* independent points, provided *N* is strictly less than $(1/2+o(1))\,(n\ln n)\cdot \operatorname {vol}(\mathbb {T})/\operatorname {vol}(K)$.

Recall that the covering result of Rogers [15] holds for all convex bodies. A natural question is whether the Euclidean ball in Theorem 1.2(i) can be replaced by an arbitrary convex body. The following result shows that this is not the case.

### Theorem 1.5

Let δ>0 be a real number. Let $C_{n}:=[-1/2,1/2]^n$ be the unit cube in $\mathbb {R}^{n}$ (of volume 1) and $\mathbb {T}$ be a packing torus of $C_n-C_n$ (which is equal to $2 C_{n}$). Suppose that $\textbf{X} \subseteq \mathbb {T}$ is sampled from the Poisson point process with intensity

$$\begin{aligned} \rho :=n \ln n - (1+ \delta ) n \ln \ln n . \end{aligned}$$

Then the probability that $\mathbb {T} \subseteq \textbf{X} + C_n$ tends to 0 as $n\rightarrow \infty$.

Similarly as in Remark 1.4, the conclusion of Theorem 1.5 also holds if we sample fewer than $(1+o(1))n\ln n\cdot \operatorname {vol}(\mathbb {T})$ uniform points in $\mathbb {T}$. Also, the proof of Rogers [15] can be adapted to show the constant 1 in front of nlnn in Theorem 1.5 is best possible. Thus, rather surprisingly, an *n*-dimensional cube is a “worst” convex body for covering by random translations.

In the next section, we present the proof of Theorem 1.2. In Section 3, we give the proofs of Theorems 1.3 and 1.5.

## Proof of Theorem 1.2

In this section, we present the proof of Theorem 1.2.

We will use the following standard estimate for the tail of the Poisson distribution, which can be found, for example, in [13, Theorem 5.4].

### Lemma 2.1

Suppose that $\textbf{X} \sim \textrm{Pois}(\lambda )$. Then the following statements hold.

(i) For every σ>0,
  $$\begin{aligned} \mathbb {P}\big [\, \textbf{X} \ge (1+\sigma ) \lambda \, \big ] \le \textrm{e}^{-\lambda } \left( \frac{\textrm{e}\lambda }{(1+\sigma ) \lambda }\right) ^{(1+\sigma ) \lambda } = \left( \frac{\textrm{e}^{\sigma }}{(1+\sigma )^{1+\sigma }}\right) ^{\lambda }. \end{aligned}$$
(ii) For every $\sigma \in (0,1)$,
  $$\begin{aligned} \mathbb {P}\big [\, \textbf{X} \le (1-\sigma ) \lambda \, \big ] \le \left( \frac{\textrm{e}^{-\sigma }}{(1-\sigma )^{1-\sigma }}\right) ^{\lambda }. \end{aligned}$$

We will also use the following simple geometric result.

### Lemma 2.2

Let δ,r>0 be real numbers and let $n\rightarrow \infty$. Suppose that $\textbf{x}_{1}, \textbf{x}_{2} \in \mathbb {R}^{n}$ are two points satisfying $\Vert \textbf{x}_{1}-\textbf{x}_{2}\Vert \le r n^{-{1}/{2} - \delta }$. Then

$$\begin{aligned} \operatorname {vol}\big ( B_{r}^{n}(\textbf{x}_{1}) \setminus B_{r}^{n}(\textbf{x}_{2}) \big ) \le \frac{1+o(1)}{\sqrt{2 \pi }}\, n^{-\delta } \cdot \operatorname {vol}\big (B_{r}^{n}\big ). \end{aligned}$$

### Proof of Lemma 2.2

Let $\textbf{x}_{1}, \textbf{x}_{2} \in \mathbb {R}^{n}$ be two points such that $\Vert \textbf{x}_{1}-\textbf{x}_{2}\Vert \le r n^{-{1}/{2} - \delta }$. Using the facts

$$\begin{aligned} \operatorname {vol}(B_{r}^{n}) = \frac{\pi ^{n/2}}{\Gamma \left( \frac{n}{2} + 1\right) }\, r^n \quad \text {and}\quad \frac{\Gamma \left( \frac{n}{2}+1\right) }{\Gamma \left( \frac{n-1}{2}+1\right) } =(1+o(1)) \sqrt{\frac{n}{2}}, \end{aligned}$$

we obtain

3 $$\begin{aligned} \frac{\operatorname {vol}\big (B_{r}^{n-1}\big )}{\operatorname {vol}\big (B_{r}^{n}\big )} = \frac{1}{r \sqrt{\pi }} \frac{\Gamma \left( \frac{n}{2}+1\right) }{\Gamma \left( \frac{n-1}{2}+1\right) } = \frac{1+o(1)}{r \sqrt{2 \pi }} \sqrt{n}. \end{aligned}$$

Let $\textrm{conv}(X)$ denote the convex hull of a set $X\subseteq \mathbb {R}^n$. It follows from (3) that

$$\begin{aligned} \operatorname {vol}\big ( B_{r}^{n}(\textbf{x}_{1}) \setminus B_{r}^{n}(\textbf{x}_{2}) \big )&= \operatorname {vol}\big ( B_{r}^{n}(\textbf{x}_{1}) \cup B_{r}^{n}(\textbf{x}_{2}) \big ) - \operatorname {vol}\big ( B_{r}^{n}(\textbf{x}_{2}) \big ) \\&\le \operatorname {vol}\big ( \textrm{conv}\big (B_{r}^{n}(\textbf{x}_{1}) \cup B_{r}^{n}(\textbf{x}_{2}) \big ) \big ) - \operatorname {vol}\big ( B_{r}^{n}(\textbf{x}_{2}) \big ) \\&= \Vert \textbf{x}_{1}-\textbf{x}_{2}\Vert \cdot \operatorname {vol}\big (B_{r}^{n-1}\big ) \\&\le \frac{r}{ n^{1/2 + \delta }}\, \frac{1+o(1)}{r \sqrt{2 \pi }}\, \sqrt{n} \cdot \operatorname {vol}\big (B_{r}^{n}\big ) = \frac{1+o(1)}{\sqrt{2 \pi }}\, n^{-\delta } \cdot \operatorname {vol}\big (B_{r}^{n}\big ), \end{aligned}$$

as desired. □

We are now ready to present the proof of Theorem 1.2.

In this result as well as in Theorems 1.3 and 1.5, we work inside a packing torus $\mathbb {T}=\mathbb {R}^n/\Lambda$ of K-K (where $K=B_1^n$ in Theorem 1.2). Note that the projection $\mathbb {R}^n\rightarrow \mathbb {T}$ is injective on any translate $K+\textbf{p}\subseteq \mathbb {R}^n$. Indeed, if we had $\textbf{k}+\textbf{p}+\textbf{x}=\mathbf {k'}+\textbf{p}$ for some $\textbf{x}\in \Lambda$ and distinct $\textbf{k},\mathbf {k'}\in K$ then $K\cap (K+\textbf{x})$ would contain $\mathbf {k'}=\textbf{k}+\textbf{x}$, a contradiction to our assumption that Λ is a packing lattice for K-K (which contains a copy of *K*). In particular, the measure of a subset of any translate of *K* is the same, regardless whether we compute it inside $\mathbb {R}^n$ or inside $\mathbb {T}$, justifying our usage of the same notation $\operatorname {vol}(\cdot )$ in both cases. Also, the distance between $\textbf{x},\textbf{y}\in \mathbb {T}$ is defined as the distance between their preimage sets in $\mathbb {R}^n$ (and still denoted by $\Vert \textbf{x}-\textbf{y}\Vert$). Note that, in the setting of Theorem 1.2, the projection is also an isometry on each radius-1 ball in $\mathbb {R}^n$. We will use these facts a number of times in the forthcoming proofs without explicitly mentioning them.

### Proof of Theorem 1.2

Fix δ>0, which we may assume to be small. Let $\beta = \beta (\delta ) \in (0, 1)$ be a real number satisfying

4 $$\begin{aligned} \left( \frac{\textrm{e}^{\beta -1}}{\beta ^\beta }\right) ^{1+\frac{\delta }{2}} \le \textrm{e}^{-1}. \end{aligned}$$

Since $\lim _{x \rightarrow 0} \frac{\textrm{e}^{x-1}}{x^x} = \textrm{e}^{-1}$ with convergence from above, such a real $\beta \in (0,1)$ exists.

Let *n* be a sufficiently large integer. Define

$$\begin{aligned} \varepsilon :=\frac{1}{n \ln n} \quad \text {and}\quad \mu :=n^{-\frac{1}{2} - \frac{\delta }{4}}. \end{aligned}$$

Let $\mathbb {T}$ be a packing torus of $B_{2}^{n}$ with volume at most $M^n \nu _{n}$. Let $P_{\varepsilon } \subseteq \mathbb {T}$ and $P_{\mu } \subseteq \mathbb {T}$ be a maximal $B_{\varepsilon /2}^{n}$-packing and a maximal $B_{\mu /2}^{n}$-packing of $\mathbb {T}$, respectively. By maximality, $P_{\varepsilon }$ forms a $B_\varepsilon ^n$-covering of $\mathbb {T}$ and $P_{\mu }$ forms a $B_\mu ^n$-covering of $\mathbb {T}$.

Also, we have the trivial upper bounds

5 $$\begin{aligned} |P_\varepsilon | \le \frac{\textrm{vol}(\mathbb {T})}{\textrm{vol}(B^n_{\varepsilon /2})} \le \frac{M^n\nu _n}{(\varepsilon /2)^n\nu _n}=M^n(\varepsilon /2)^{-n} \quad \text {and}\quad |P_{\mu }| \le M^n \left( \frac{\mu }{2}\right) ^{-n}. \end{aligned}$$

Fix a map $\phi :P_{\varepsilon } \rightarrow P_{\mu }$ such that, for every $\textbf{x} \in P_{\varepsilon }$,

$$\begin{aligned} \Vert \textbf{x}-\phi (\textbf{x})\Vert \le \mu . \end{aligned}$$

Note that such a map exists since $P_{\mu }$ is a $B_{\mu }^{n}$-covering.

Let $\textbf{X}$ be a set sampled from the Poisson point process on $\mathbb {T}$ with intensity

6 $$\begin{aligned} \rho :=\left( \frac{1}{2} + \delta \right) \frac{n \ln n}{\nu _{n}}. \end{aligned}$$

### Claim 2.3

The probability that $|\textbf{X}| \le (1+\delta ) \rho \cdot \operatorname {vol}(\mathbb {T})$ tends to 1 as $n \rightarrow \infty$.

### Proof of Claim 2.3

Since $\mathbb {T}$ is a packing torus of $B_{2}^{n}$, we trivially have $\operatorname {vol}(\mathbb {T}) \ge \operatorname {vol}(B_{2}^{n}) = 2^{n} \nu _{n}$. The random variable $|\textbf{X}|$ has the Poisson distribution with mean $\rho \cdot \operatorname {vol}(\mathbb {T}) \ge 2^{n-1} n \ln n$. It follows from Lemma 2.1 and the crude inequality $\frac{\textrm{e}^{x}}{(1+x)^{1+x}} \le \textrm{e}^{-x^2/10}$ for x∈[0,1] that

$$\begin{aligned} \mathbb {P}\left[ \,|\textbf{X}| \ge (1+\delta ) \rho \cdot \operatorname {vol}(\mathbb {T})\,\right] \le \left( \frac{\textrm{e}^{\delta }}{(1+\delta )^{1+\delta }}\right) ^{\rho \cdot \operatorname {vol}(\mathbb {T})} \le \textrm{exp}\left( -\frac{\delta ^2}{10} \cdot 2^{n-1} n \ln n\right) , \end{aligned}$$

which goes to 0 as $n \rightarrow \infty$. □

For every point $\textbf{y} \in \mathbb {T}$ and d≥0, let

$$\begin{aligned} N_{d}(\textbf{y}) :=B_{d}^{n}(\textbf{y}) \cap \textbf{X} = \left\{ \textbf{x} \in \textbf{X} :\Vert \textbf{x}-\textbf{y}\Vert \le d \right\} . \end{aligned}$$

We say that a point $\textbf{y}\in \mathbb {T}$ is *saturated* if

$$\begin{aligned} |N_{1-\varepsilon }(\textbf{y})| \ge \frac{\beta }{2}\, n \ln n. \end{aligned}$$

Define the events $E_{1}$, $E_{2}$ and $E_{3}$ as follows:

- $E_{1}$: every point in $P_{\mu }$ is saturated.
- $E_{2}$: $|N_{1+\varepsilon }(\textbf{z})| \le \left( \xi + 10\delta \right) n \ln n$ for every $\textbf{z} \in P_{\varepsilon }$, where ξ=1.79556... is the unique real root of (1).
- $E_{3}$: for each $\textbf{z} \in P_{\varepsilon }$, either $|N_{1-\varepsilon }(\textbf{z})|>0$, or $\phi (\textbf{z})$ is not saturated.

Next, we show that each of these events holds with high probability.

### Claim 2.4

We have $\min \left\{ \,\mathbb {P}[E_1],\,\mathbb {P}[E_2],\,\mathbb {P}[E_3]\,\right\} \rightarrow 1$ as $n\rightarrow \infty$.

### Proof of Claim 2.4

First, we prove that $\mathbb {P}(E_1) \rightarrow 1$. Fix an arbitrary point $\textbf{y} \in P_{\mu }$. The random variable $|N_{1-\varepsilon }(\textbf{y})|$ has the Poisson distribution with mean

$$\begin{aligned} \rho \cdot \operatorname {vol}(B_{1-\varepsilon }^{n}) = \left( \frac{1}{2} + \delta \right) \frac{n \ln n}{\nu _{n}} \cdot \operatorname {vol}(B_{1-\varepsilon }^{n}) = \left( \frac{1}{2} + \delta \right) (1-\varepsilon )^{n} n \ln n. \end{aligned}$$

Recall that $\varepsilon =(n\ln n)^{-1}$. Since 0<δ<1 is fixed while *n* is sufficiently large, it holds that

$$\begin{aligned} \left( \frac{1}{2}+\delta \right) (1-\varepsilon )^{n} \ge \frac{1}{2} \quad \text {and}\quad \frac{2}{2+\delta } \left( \frac{1}{2}+\delta \right) (1-\varepsilon )^{n} \ge \frac{1}{2} + \frac{\delta }{3}. \end{aligned}$$

Applying Lemma 2.1  with $\sigma = 1 - \beta$, and using (4), we obtain

$$\begin{aligned}&\mathbb {P}\left[ \,|N_{1-\varepsilon }(\textbf{y})| \le \frac{\beta }{2}\, n \ln n\,\right] \\&\le \mathbb {P}\left[ \,|N_{1-\varepsilon }(\textbf{y})| \le \left( 1 - (1-\beta ) \right) \left( \frac{1}{2} + \delta \right) (1-\varepsilon )^{n} n \ln n\, \right] \\&\le \left( \frac{\textrm{e}^{\beta -1}}{\beta ^{\beta }}\right) ^{\left( \frac{1}{2} + \delta \right) (1-\varepsilon )^{n} n \ln n} \\&\le \textrm{e}^{-\frac{2}{2+\delta } \cdot \left( \frac{1}{2} + \delta \right) (1-\varepsilon )^{n} n \ln n} \le \textrm{e}^{-\left( \frac{1}{2} + \frac{\delta }{3}\right) n \ln n} = n^{-\left( \frac{1}{2} + \frac{\delta }{3}\right) n}. \end{aligned}$$

Combining this with (5), we obtain

$$\begin{aligned} \mathbb {P}[E_1]&\ge 1 - \sum _{\textbf{y} \in P_{\mu }} \mathbb {P}\left[ \,|N_{1-\varepsilon }(\textbf{y})| \le \frac{\beta }{2}\, n \ln n\,\right] \\&\ge 1 - M^{n} \left( \frac{\mu }{2}\right) ^{-n} n^{-\left( \frac{1}{2} + \frac{\delta }{3}\right) n} = 1 - \left( \frac{n^{\delta /12}}{2M}\right) ^{-n} \rightarrow 1 \quad \text {as}\quad n \rightarrow \infty , \end{aligned}$$

as desired.

We now consider $E_2$. Fix an arbitrary point $\textbf{z} \in P_{\varepsilon }$. As in the preceding proof, the random variable $|N_{1+\varepsilon }(\textbf{z})|$ has the Poisson distribution with mean

$$\begin{aligned} \rho \cdot \operatorname {vol}(B_{1+\varepsilon }^{n}) = \left( \frac{1}{2} + \delta \right) \frac{n \ln n}{\nu _{n}} \cdot \operatorname {vol}(B_{1+\varepsilon }^{n}) = \left( \frac{1}{2} + \delta \right) (1+\varepsilon )^{n} n \ln n. \end{aligned}$$

Let $\xi _0 :=2\xi - 1 = 2.59112...\,$. Since ξ is the root of (1), a straightforward calculation shows that $\xi _{0}$ is the unique real root of

7 $$\begin{aligned} \frac{\textrm{e}^{x}}{(1+x)^{1+x}} = \frac{1}{\textrm{e}^2}. \end{aligned}$$

Also, we have

$$\begin{aligned} \left( \frac{1}{2} + \delta \right) (1+\varepsilon )^{n} \le \frac{1}{2} + 2 \delta . \end{aligned}$$

Since ξ=1.79... is at most 5/2, we obtain

$$\begin{aligned} \frac{\xi + 10 \delta }{(1/2+\delta ) (1+\varepsilon )^{n}} - (1+\xi _0) \ge \frac{\xi + 10 \delta }{1/2 + 2\delta } - 2\xi = \frac{4(5-2\xi )\delta }{1+4\delta } \ge 0. \end{aligned}$$

Now applying Lemma 2.1 with $\sigma = \xi _0$, and using (7), we obtain

$$\begin{aligned}&\mathbb {P}\left[ \,|N_{1+\varepsilon }(\textbf{z})| \ge (\xi + 10\delta ) n \ln n\,\right] \\&\le \mathbb {P}\left[ \, |N_{1+\varepsilon }(\textbf{z})| \ge (1 + \xi _0) \left( \frac{1}{2} + \delta \right) (1+\varepsilon )^{n} n \ln n\, \right] \\&\le \left( \frac{\textrm{e}^{\xi _0}}{(1+\xi _0)^{1+\xi _0}}\right) ^{\left( \frac{1}{2} + \delta \right) (1+\varepsilon )^{n} n \ln n} \\&= \textrm{e}^{-2 \left( \frac{1}{2} + \delta \right) (1+\varepsilon )^{n} n \ln n} \le \textrm{e}^{-(1+2\delta ) n \ln n} = n^{-(1+2\delta )n}. \end{aligned}$$

Combining it with (5), we obtain

$$\begin{aligned} \mathbb {P}[E_2]&\ge 1 - \sum _{\textbf{z} \in P_{\varepsilon }} \mathbb {P}\left[ \,|N_{1+\varepsilon }(\textbf{z})| \ge (\xi + 10\delta ) n \ln n\,\right] \\&\ge 1 - M^n \left( \frac{\varepsilon }{2}\right) ^{-n} n^{-(1+2\delta )n} \\&= 1 - \left( \frac{n^{1+2\delta }}{2M n \ln n}\right) ^{-n} = 1 - \left( \frac{n^{2\delta }}{2M \ln n}\right) ^{-n} \rightarrow 1 \quad \text {as}\quad n \rightarrow \infty , \end{aligned}$$

as desired.

Finally, we consider $E_3$. For a point $\textbf{z} \in P_{\varepsilon }$, let $B_{\textbf{z}}$ denote the (bad) event that

$$\begin{aligned} |N_{1-\varepsilon }(\textbf{z})|=0 \text { \quad and\quad } \phi (\textbf{z}) \in P_{\mu } \text { is saturated}. \end{aligned}$$

Next, we analyze the probability that $B_{\textbf{z}}$ occurs.

Let $S :=B_{1-\varepsilon }^{n}\big (\phi (\textbf{z})\big ) \setminus B_{1-\varepsilon }^{n}(\textbf{z})$. By the definition of ϕ, we have

$$\begin{aligned} \Vert \textbf{z}-\phi (\textbf{z})\Vert \le \mu = n^{-\frac{1}{2} - \frac{\delta }{4}} \le (1-\varepsilon ) n^{-\frac{1}{2} - \frac{\delta }{8}}. \end{aligned}$$

So it follows from Lemma 2.2 that

8 $$\begin{aligned} \operatorname {vol}(S) \le \frac{1+o(1)}{\sqrt{2 \pi }}\, n^{-\delta /8} \cdot \operatorname {vol}\big (B_{1-\varepsilon }^{n}\big ) \le n^{-\delta /8} \nu _{n}. \end{aligned}$$

Since the random variable $|S \cap \textbf{X}|$ has the Poisson distribution with mean

$$\begin{aligned} \rho \cdot \operatorname {vol}(S) = \left( \frac{1}{2}+\delta \right) \frac{n \ln n}{\nu _{n}}\, \operatorname {vol}(S) \le \left( \frac{1}{2}+\delta \right) n^{1-\frac{\delta }{8}} \ln n \ll \frac{\beta }{2}\, n \ln n, \end{aligned}$$

it follows from Lemma 2.1 and (6) that

$$\begin{aligned} \mathbb {P}\left[ \,|S\cap \textbf{X}| \ge \frac{\beta }{2}\, n \ln n\,\right]&\le \textrm{e}^{- \rho \cdot \operatorname {vol}(S)} \left( \frac{\textrm{e}\rho \cdot \operatorname {vol}(S)}{(\beta /2) n \ln n}\right) ^{\frac{\beta }{2}\, n \ln n}\\&\le \left( \frac{\beta n \ln n}{2 \textrm{e}\rho \cdot \operatorname {vol}(S)}\right) ^{-\frac{\beta }{2}\, n \ln n} \le \left( \frac{\beta \nu _n }{2 \textrm{e}\cdot \operatorname {vol}(S)}\right) ^{-\frac{\beta }{2}\, n \ln n}. \end{aligned}$$

Since $\beta = \beta (\delta )> 0$ while *n* is sufficiently large, we have that

$$\begin{aligned} \frac{\beta }{2\textrm{e}} \ge n^{-\frac{\delta }{40}}. \end{aligned}$$

Combining this with (8), we obtain

$$\begin{aligned} \mathbb {P}\left[ \,|S\cap \textbf{X}| \ge \frac{\beta }{2}\, n \ln n\,\right] \le \left( n^{-\frac{\delta }{40}} \frac{\nu _{n}}{n^{-\delta /8} \nu _{n}}\right) ^{-\frac{\beta }{2}\, n \ln n} = n^{-\frac{\beta \delta }{20}\, n \ln n}. \end{aligned}$$

By definition, $B_{\textbf{z}}$ occurs if and only if

$$\begin{aligned} |N_{1-\varepsilon }(\textbf{z})| = 0 \quad \text {and}\quad |N_{1-\varepsilon }(\phi (\textbf{z}))| \ge \frac{\beta }{2}\, n \ln n, \end{aligned}$$

which implies that all points in the set $N_{1-\varepsilon }(\phi (\textbf{z}))$ lie within the region *S*. Therefore, for any given $\textbf{z}\in P_{\varepsilon }$, it holds that

$$\begin{aligned} \mathbb {P}[B_{\textbf{z}}] \le \mathbb {P}\left[ \,|S \cap \textbf{X}| \ge \frac{\beta }{2} n \ln n\,\right] \le n^{-\frac{\beta \delta }{20} n \ln n}. \end{aligned}$$

Combining it with (5), we obtain

$$\begin{aligned} \mathbb {P}[E_3] \ge 1 - \sum _{\textbf{z} \in P_{\varepsilon }} \mathbb {P}[B_{\textbf{z}}]&\ge 1 - M^n \left( \frac{\varepsilon }{2}\right) ^{-n} n^{-\frac{\beta \delta }{20}\, n \ln n} \\&= 1 - \textrm{exp}\left( n \ln (2M\, n \ln n) - \frac{\beta \delta }{20} n \ln ^2 n \right) \rightarrow 1 \,\text {as}\, n \rightarrow \infty . \end{aligned}$$

This completes the proof of Claim 2.4. □

Thus there is an outcome $\textbf{X}$ of size at most $(1+\delta ) \rho \cdot \operatorname {vol}(\mathbb {T})$ for which all of the events $E_1$, $E_2$, and $E_3$ occur. Fix any such $\textbf{X}$.

### Claim 2.5

The set $\textbf{X}$ is a unit ball covering of $\mathbb {T}$. Moreover,

$$\begin{aligned} \vartheta (\textbf{X},\mathbb {T}) \le \left( \frac{1}{2} + 2\delta \right) n \ln n \quad \text {and}\quad \mu (\textbf{X},\mathbb {T}) \le \left( \xi + 10 \delta \right) n \ln n. \end{aligned}$$

### Proof of Claim 2.5

First, we show that $\textbf{X}$ is a unit ball covering of $\mathbb {T}$. Fix an arbitrary point $\textbf{w} \in \mathbb {T}$. Since $P_{\varepsilon }$ is a $B_{\varepsilon }^{n}$-covering of $\mathbb {T}$, there exists a point $\textbf{z} \in P_{\varepsilon }$ such that $\Vert \textbf{w}- \textbf{z}\Vert \le \varepsilon$. The fact that the event $E_1$ holds gives that the point $\phi (\textbf{z}) \in P_{\mu }$ is saturated. Combining it with the fact that the event $E_3$ holds, we conclude that there exists a point $\textbf{x} \in \textbf{X}$ such that $\Vert \textbf{z}- \textbf{x}\Vert \le 1- \varepsilon$. Therefore,

$$\begin{aligned} \Vert \textbf{w}- \textbf{x}\Vert \le \Vert \textbf{w}- \textbf{z}\Vert + \Vert \textbf{z}- \textbf{x}\Vert \le 1, \end{aligned}$$

which shows that $\textbf{X}$ is a unit ball covering of $\mathbb {T}$.

By definition, the covering density of $\textbf{X}$ is

$$\begin{aligned} \vartheta (\textbf{X},\mathbb {T}) = \sum _{\textbf{x} \in \textbf{X}} \frac{\operatorname {vol}\left( B_{1}^{n}(\textbf{x}) \cap \mathbb {T}\right) }{\operatorname {vol}(\mathbb {T})} = |\textbf{X}|\, \frac{\nu _{n}}{\operatorname {vol}(\mathbb {T})}&\le (1+\delta ) \left( \frac{1}{2} + \delta \right) \frac{n \ln n}{\nu _{n}} \operatorname {vol}(\mathbb {T})\, \frac{\nu _{n}}{\operatorname {vol}(\mathbb {T})} \\&\le \left( \frac{1}{2} + 2\delta \right) n \ln n, \end{aligned}$$

as desired.

Now suppose to the contrary that there exists a point $\textbf{u} \in \mathbb {T}$ such that

$$\begin{aligned} \mu (\textbf{X}, \textbf{u}) > (\xi + 10 \delta ) n \ln n. \end{aligned}$$

Since $P_{\varepsilon }$ is a $B_\varepsilon ^n$-covering of $\mathbb {T}$, there exists a point $\textbf{z} \in P_{\varepsilon }$ such that $\Vert \textbf{u}- \textbf{z}\Vert \le \varepsilon$. Then

$$\begin{aligned} |N_{1+\varepsilon }(\textbf{z})| \ge \mu (\textbf{X},\textbf{u}) > (\xi +10\delta )n\ln n \end{aligned}$$

which contradicts the fact that the event $E_2$ occurs.

Claim 2.5 completes the proof of Theorem 1.2. □

## Proofs of Theorems 1.3 and 1.5

In this section, we present the proofs of Theorems 1.3 and 1.5.

A convex body $K \subseteq \mathbb {R}^n$ is said to be *isotropic* if $\int _{K} \textbf{x}\, \textrm{d}\textbf{x} = 0$, $\operatorname {vol}(K) = 1$, and there exists a constant $L_K$, called the *isotropic constant* of *K*, such that for every $\textbf{y} \in \mathbb {R}^{n}$,

9 $$\begin{aligned} \int _K \langle \textbf{x}, \textbf{y} \rangle ^2 \, \textrm{d}\textbf{x} = L_K^2 \Vert \textbf{y}\Vert ^2. \end{aligned}$$

It was a long-standing open problem to determine whether $L_K$ can be bounded by an absolute constant, independent of the dimension *n*. Culminating decades of work by many researchers, the question was recently resolved affirmatively by Guan, Klartag and Lehec [10, 12].

### Theorem 3.1

(Guan [10], Klartag and Lehec [12]) There exists an absolute constant *C* such that the following holds for every $n \in \mathbb {N}$. Suppose that $K \subseteq \mathbb {R}^n$ is an isotropic convex body. Then the isotropic constant $L_{K}$ of *K* satisfies $L_{K} \le C$.

Since every convex body can be made isotropic via a suitable affine transformation (see e.g.  [3, Solution to Exercise 9.17]), it suffices to prove Theorem 1.3 for isotropic convex bodies. The following lemma will be used in the proof.

### Lemma 3.2

Suppose that $K \subseteq \mathbb {R}^{n}$ is an isotropic convex body and $\textbf{x} \in \mathbb {R}^{n}$ is a vector satisfying $\Vert \textbf{x}\Vert \ge 4 L_{K}$. Then

$$\begin{aligned} \operatorname {vol}\left( K \cap (K + \textbf{x}) \right) \le 3^{-\frac{\Vert \textbf{x}\Vert }{8L_{K}}}. \end{aligned}$$

### Proof of Lemma 3.2

Fix a vector $\textbf{x} \in \mathbb {R}^{n}$ satisfying $\Vert \textbf{x}\Vert \ge 4 L_{K}$. For every real number $w \in \mathbb {R}$, define the *slab of width w in the direction of* $\textbf{x}$ as

$$\begin{aligned} S_{\textbf{x}}(w) :=\left\{ \textbf{y} :|\langle \textbf{x}, \textbf{y} \rangle | < w\, \Vert \textbf{x}\Vert \right\} . \end{aligned}$$

Markov’s inequality applied to the random variable $\langle \textbf{x}, \textbf{y} \rangle ^2$, where $\textbf{y}$ is a uniform element of *K*, implies by (9) that

10 $$\begin{aligned} \operatorname {vol}\left( K \setminus S_{\textbf{x}}(2L_{K})\right) \le \frac{1}{4}. \end{aligned}$$

A well-known argument due to Borell [4, Lemma 3.1] gives that

11 $$\begin{aligned} \operatorname {vol}\left( K \setminus S_{\textbf{x}}(2tL_K)\right) \le \frac{\sqrt{3}}{4} \cdot 3^{-\frac{t}{2}}, \quad \text {for every}\quad t \ge 1. \end{aligned}$$

For completeness, let us sketch a proof of (11). The uniform probability measure $\mu _K$ on *K* is clearly *logarithmically concave*, meaning that the function $-\log (\textrm{d}\mu _K/\textrm{d}\mu )$ (which, in this case, is 0 on *K* and ∞ elsewhere) is convex. By a theorem of Borell [5, Theorem 3.2], $\mu _K$ satisfies the Multiplicative Brunn-Minkowski Inequality. Next, it is routine to check that, for any convex centrally symmetric *A*, it holds that

$$ \mathbb {R}^n\setminus A\supseteq \frac{2}{t+1}\,(\mathbb {R}^n\setminus tA)+\frac{t-1}{t+1}\, A. $$

Evaluating $\mu _K$ on this inclusion for the slab $A:=S_{\textbf{x}}(2L_K)$, we obtain that

$$ \operatorname {vol}(K\setminus A)\ge \mu _K\left( \frac{2}{t+1}\,(\mathbb {R}^n\setminus tA)+\frac{t-1}{t+1}\, A\right) \ge \operatorname {vol}(K\setminus tA)^{\frac{2}{t+1}}\cdot \operatorname {vol}(K\cap A)^{\frac{t-1}{t+1}}, $$

which implies (11) by (10).

In particular, by applying (11) with $t :={\Vert \textbf{x}\Vert }/(4L_{K})$ and denoting $S :=S_{\textbf{x}}\left( \Vert \textbf{x}\Vert /2\right)$ for brevity, we obtain

$$\begin{aligned} \operatorname {vol}\left( K \setminus S\right) \le \frac{\sqrt{3}}{4} \cdot 3^{-\frac{\Vert \textbf{x}\Vert }{8L_{K}}}. \end{aligned}$$

### Claim 3.3

We have that $S \cap (S + \textbf{x}) = \emptyset$.

### Proof of Claim 3.3

Suppose to the contrary that there exists $\textbf{y} \in S \cap (S + \textbf{x})$. Then $\textbf{y} \in S$ and $\textbf{y} - \textbf{x} \in S$. It follows from the definition of *S* that

$$\begin{aligned} \Vert \textbf{x}\Vert ^2 = |\langle \textbf{x}, \textbf{x} \rangle | = |\langle \textbf{y}, \textbf{x} \rangle - \langle \textbf{y} - \textbf{x}, \textbf{x} \rangle |\\ \le |\langle \textbf{y}, \textbf{x} \rangle | + |\langle \textbf{y} - \textbf{x}, \textbf{x} \rangle | < \frac{\Vert \textbf{x}\Vert ^2}{2} + \frac{\Vert \textbf{x}\Vert ^2}{2} = \Vert \textbf{x}\Vert ^2, \end{aligned}$$

a contradiction.

Let $\overline{S} :=K \setminus S$. It follows from Claim 3.3 that

$$\begin{aligned} K \cap (K + \textbf{x}) \subseteq \overline{S} \cup (\overline{S} + \textbf{x}). \end{aligned}$$

Therefore,

$$\begin{aligned} \operatorname {vol}\left( K \cap (K + \textbf{x})\right) \le \operatorname {vol}(\overline{S}) + \operatorname {vol}(\overline{S} + \textbf{x})\\ = 2 \cdot \operatorname {vol}(K \setminus S) \le 2 \cdot \frac{\sqrt{3}}{4} \cdot 3^{-\frac{\Vert \textbf{x}\Vert }{8L_{K}}} \le 3^{-\frac{\Vert \textbf{x}\Vert }{8L_{K}}}, \end{aligned}$$

which proves Lemma 3.2. □

We will also use the following standard result from probability, which can be found in e.g. [2, Theorem 4.3.1].

### Lemma 3.4

Suppose that *X* is a nonnegative, integral-valued random variable with $\mathbb {E}X>0$. Then

$$\begin{aligned} \mathbb {P}[\,X = 0\,] \le \frac{\textrm{Var}[X]}{\mathbb {E}[X]^2}, \end{aligned}$$

where $\textrm{Var}[X]$ and $\mathbb {E}[X]$ denote respectively the variance and the expectation of *X*.

Before proving Theorem 1.3, we first establish the following result.

### Theorem 3.5

Let $n\rightarrow \infty$. Let $K \subseteq \mathbb {R}^{n}$ be an isotropic convex body and let $\mathbb {T}$ be a packing torus of K-K. Suppose that $P \subseteq \mathbb {T}$ is a $B_{1/2}^{n}$-packing and $\omega (n)\rightarrow \infty$ is a function such that

$$\begin{aligned} \rho :=\ln |P| - n \big ( \ln \ln \ln |P| + \omega (n) \big ) \end{aligned}$$

tends to infinity. Let $\textbf{X} \subseteq \mathbb {T}$ be a set sampled from the Poisson point process on $\mathbb {T}$ with intensity ρ. Then $\mathbb {P}\left[ \,P \subseteq K + \textbf{X}\,\right]$ tends to 0.

### Proof of Theorem 3.5

For every point $\textbf{p} \in P$, let $B_{\textbf{p}}$ be the characteristic function of the event that $\textbf{p} \notin \textbf{X} + K$, that is,

$$\begin{aligned} B_{\textbf{p}} = {\left\{ \begin{array}{ll} 1, & \quad \text {if}\quad \textbf{p} \notin \textbf{X} + K, \\ 0, & \quad \text {if}\quad \textbf{p} \in \textbf{X} + K. \end{array}\right. } \end{aligned}$$

Since $\operatorname {vol}(K) = 1$ (by the definition of isotropic), the random variable $|\textbf{X} \cap (\textbf{p} - K)|$ has the Poisson distribution with mean $\rho \cdot \operatorname {vol}(\textbf{p} - K) = \rho$. Therefore, $\mathbb {E}[B_{\textbf{p}}] = \textrm{e}^{-\rho }$. It follows that the sum $B :=\sum _{\textbf{p} \in P}B_{\textbf{p}}$ satisfies

12 $$\begin{aligned} \mathbb {E}[B] = |P|\, \textrm{e}^{-\rho } = \textrm{e}^{n \left( \ln \ln \ln |P| + \omega (n) \right) } \rightarrow \infty \quad \text {as}\quad n \rightarrow \infty . \end{aligned}$$

Note that *B* is the number of points in *P* that are not covered by $\textbf{X} + K$. We will use Lemma 3.4 (that is, the second moment method) to show that $\mathbb {P}[\,B = 0\,]\rightarrow 0$ as $n \rightarrow \infty$.

Let $\Delta :=8 L_K (f_n + \log _3 \rho )$, where $f_n \rightarrow \infty$ very slowly (as $n \rightarrow \infty$). Let

$$\begin{aligned} \Sigma _1 :=\sum _{\begin{array}{c} \textbf{p},\textbf{p}'\in P\\ \Vert \textbf{p} - \textbf{p}'\Vert \le \Delta \end{array}} \mathbb {E}[B_\textbf{p} B_{\textbf{p}'}] \quad \text {and}\quad \Sigma _2 :=\sum _{\begin{array}{c} \textbf{p},\textbf{p}'\in P\\ \Vert \textbf{p} - \textbf{p}'\Vert > \Delta \end{array}} \left( \mathbb {E}[B_\textbf{p} B_{\textbf{p}'}] - \mathbb {E}[B_\textbf{p}]\, \mathbb {E}[B_{\textbf{p}'}] \right) . \end{aligned}$$

Since *P* is a $B_{1/2}^{n}$-packing of $\mathbb {T}$, for every point $\textbf{p} \in P$ the number of points in *P* satisfying $\Vert \textbf{p} - \textbf{p}'\Vert \le \Delta$ is at most

$$\begin{aligned} \frac{\operatorname {vol}\big (B_{\Delta +1/2}^{n}(\textbf{p})\big )}{\operatorname {vol}\big (B_{1/2}^{n}\big )} = \left( \frac{\Delta + 1/2}{1/2}\right) ^{n} \le \left( 4\Delta \right) ^{n}. \end{aligned}$$

Notice that

$$\begin{aligned} \ln&\left( \frac{\left( 32 L_K (f_n + \log _3 \rho ) \right) ^{n}}{|P| \textrm{e}^{-\rho }}\right) \\&= n \ln \left( 32 L_K (f_n + \log _3 \rho )\right) - n \big ( \ln \ln \ln |P| + \omega (n) \big ) \\&= n \ln \left( f_n + \frac{\ln \rho }{\ln 3}\right) + n \ln (32 L_K) - n \big ( \ln \ln \ln |P| + \omega (n) \big ) \\&\le n \ln \ln \rho + n \ln (32 L_K) - n \big ( \ln \ln \ln |P| + \omega (n) \big ) \le n \big ( \ln (32 L_K) - \omega (n) \big ) \end{aligned}$$

which, by Theorem 3.1, tends to -∞ as $n \rightarrow \infty$. Therefore, by (12),

13 $$\begin{aligned} \Sigma _1 \le \sum _{\textbf{p} \in P} \mathbb {E}[B_{\textbf{p}}] \left( 4\Delta \right) ^{n} = \mathbb {E}[B] \left( 4\Delta \right) ^{n} = \mathbb {E}[B]^2 \frac{\left( 32 L_K (f_n + \log _3 \rho ) \right) ^{n}}{|P|\, \textrm{e}^{-\rho }} = o\left( \mathbb {E}[B]^{2}\right) . \end{aligned}$$

Next, we consider $\Sigma _2$. Fix two points $\textbf{p},\textbf{p}' \in P$ at distance at least Δ on the torus $\mathbb {T}$. Note that $B_{\textbf{p}} B_{\textbf{p}'}$ is 1 if and only if $\textbf{X}$ does not intersect $(\textbf{p} - K) \cup (\textbf{p}' - K)$.

Hence, the number of points of $\textbf{X}$ in this set has the Poisson distribution with mean

$$\begin{aligned} \rho \cdot \operatorname {vol}\left( (\textbf{p} - K) \cup (\textbf{p}' - K)\right)&= \rho \cdot \operatorname {vol}\left( (K - \textbf{p}) \cup (K - \textbf{p}')\right) \\&= \rho \left( \operatorname {vol}(K - \textbf{p}) + \operatorname {vol}(K - \textbf{p}') - \operatorname {vol}\left( (K - \textbf{p}) \cap (K - \textbf{p}')\right) \right) \\&= 2\rho - \rho \cdot \operatorname {vol}\left( K \cap (K + \textbf{p} - \textbf{p}')\right) . \end{aligned}$$

Thus we have that

14 $$\begin{aligned} \mathbb {E}[B_{\textbf{p}} B_{\textbf{p}'}]&= \textrm{exp}\left( -\rho \cdot \operatorname {vol}\left( (\textbf{p} - K) \cup (\textbf{p}' - K)\right) \right) \nonumber \\&= \textrm{exp}\left( - 2\rho + \rho \cdot \operatorname {vol}\left( K \cap (K + \textbf{p} - \textbf{p}')\right) \right) . \end{aligned}$$

Observe that there is at most one vector $\textbf{q}\in \mathbb {R}^n$ that projects to $\textbf{p}-\textbf{p}'$ with $K\cap (K+\textbf{q})\not =\emptyset$. Indeed, if $\textbf{q}\not =\textbf{q}'$ contradict this, satisfying $\textbf{a}=\textbf{b}+\textbf{q}$ and $\textbf{a}'=\textbf{b}'+\textbf{q}'$ for some $\textbf{a},\textbf{b},\textbf{a}',\textbf{b}'$ in $K\subseteq \mathbb {R}^n$ then

$$ (\textbf{a}-\textbf{b})-(\textbf{a}'-\textbf{b}')=\textbf{q}-\textbf{q}'\in \Lambda \setminus \{\textbf{0}\}, $$

a contradiction to our assumption that Λ is a packing lattice for K-K. Furthermore, for every $\textbf{q}\in \mathbb {R}^n$ that projects to $\textbf{p}-\textbf{p}'$, it holds that $\Vert \textbf{q}\Vert \ge \Delta \ge 4 L_{K}$. Thus the upper bound of Lemma 3.2 applies also inside the torus and gives that

$$\begin{aligned} \operatorname {vol}\left( K \cap (K + \textbf{p} - \textbf{p}')\right) \le 3^{-\frac{\Vert \textbf{p} - \textbf{p}'\Vert }{8L_{K}}} \le 3^{-\frac{\Delta }{8L_{K}}} = 3^{- f_n - \log _{3} \rho } = \rho ^{-1} 3^{-f_n}. \end{aligned}$$

Combining it with (14), we obtain

15 $$\begin{aligned} \Sigma _2&\le |P|^{2} \left( \textrm{exp}\left( -2\rho + 3^{-f_n}\right) - \textrm{exp}(-2\rho ) \right) \nonumber \\&= |P|^{2} \textrm{e}^{-2\rho } \left( \textrm{exp}\left( 3^{-f_n}\right) - 1\right) = \mathbb {E}[B]^{2} \left( \textrm{exp}\left( 3^{-f_n}\right) - 1\right) = o\left( \mathbb {E}[B]^{2}\right) . \end{aligned}$$

Combining (13), (15), and Lemma 3.4, we obtain

$$\begin{aligned} \mathbb {P}[\,B = 0\,] \le \frac{\textrm{Var}[B]}{\mathbb {E}[B]^{2}} = \frac{1}{\mathbb {E}[B]^{2}} \sum _{\textbf{p}, \textbf{p}' \in P} \left( \mathbb {E}[B_\textbf{p} B_{\textbf{p}'}] - \mathbb {E}[B_\textbf{p}]\, \mathbb {E}[B_{\textbf{p}'}] \right) \le \frac{\Sigma _1 + \Sigma _2}{\mathbb {E}[B]^{2}} = o\left( 1\right) . \end{aligned}$$

This completes the proof of Theorem 3.5. □

We are now ready to present the proof of Theorem 1.3. We will use the following estimate, which follows from the fact that $\Gamma (x+1) = (1 + o(1)) \sqrt{2\pi x}\left( x/\textrm{e}\right) ^{x}$ as $x\rightarrow \infty$ (see e.g. [7]):

16 $$\begin{aligned} \nu _{n} = \frac{\pi ^{\frac{n}{2}}}{\Gamma \left( \frac{n}{2} + 1\right) } = \frac{\pi ^{\frac{n}{2}}}{(1 + o(1)) \sqrt{\pi n}\left( \frac{n}{2\textrm{e}}\right) ^{\frac{n}{2}}} = (1 + o(1)) \frac{(2\pi \textrm{e})^{\frac{n}{2}}}{\sqrt{\pi n}} n^{-\frac{n}{2}}. \end{aligned}$$

### Proof of Theorem 1.3

By applying an affine transformation to *K* (and to the lattice defining the torus $\mathbb {T}$), we can assume that $K \subseteq \mathbb {R}^{n}$ is an isotropic body. In particular, $\operatorname {vol}(K) = 1$ by definition. By monotonicity, it suffices to prove Theorem 1.3 for some $\rho \ge n\ln n/2 - (1+o(1)) n \ln \ln n$. Let $\mathbb {T}$ be a packing torus of K-K, and let *P* be a maximal $B_{1/2}^{n}$-packing of $\mathbb {T}$.

Since *P* is maximal, it is a unit ball covering of $\mathbb {T}$. Therefore, by (16),

17 $$\begin{aligned} \ln |P| \ge \ln \left( \frac{\operatorname {vol}(\mathbb {T})}{\nu _{n}} \right)&= \ln \left( (1+o(1))\frac{\operatorname {vol}(\mathbb {T})\cdot \sqrt{\pi n}}{(2\pi \textrm{e})^{\frac{n}{2}}} n^{\frac{n}{2}} \right) \nonumber \\&\ge \frac{n}{2} \ln n + \ln \left( \operatorname {vol}(\mathbb {T})\right) +n \ln \left( \frac{1}{\sqrt{2 \pi \textrm{e}}}\right) + \ln \left( \frac{\sqrt{\pi n}}{2}\right) . \end{aligned}$$

Let $g_n(t):=t-n\ln \ln t$, and note that this function is non-decreasing when $t\ln t\ge n$. In particular, this holds when *t* is at least the right-hand side of (17). Hence, by (17) and $\textrm{vol}(\mathbb {T}) \ge \textrm{vol}(K-K) \ge$1, we obtain

$$\ln |P|-n\ln \ln \ln |P| \ge \frac{n}{2}\ln n-(1+o(1))n\ln \ln n.$$

Let ω(n) be a function tending to infinity sufficiently slowly, with $\omega (n) = o(\ln \ln n)$. Let $\textbf{X}$ be a random set sampled from the Poisson point process on $\mathbb {T}$ with intensity

$$\begin{aligned} \rho :=\ln |P| - n \big (\ln \ln \ln |P| + \omega (n) \big ) \ge \frac{n}{2}\ln n - (1+o(1)) n \ln \ln n. \end{aligned}$$

It follows from Theorem 3.5 that

$$\begin{aligned} \mathbb {P}\left[ \,\mathbb {T} \subseteq \textbf{X} + K\,\right] \le \mathbb {P}\left[ \,P \subseteq \textbf{X} + K\,\right] = o(1), \end{aligned}$$

as desired. □

The proof of Theorem 1.5 is similar to that of Theorem 1.3 and will follow from the following counterpart of Theorem 3.5.

### Theorem 3.6

Let $C_{n} = [-1/2,\, 1/2]^{n}$, and let $\mathbb {T}$ be a packing torus of $2 C_n$. Let $P \subseteq \mathbb {T}$ be an $S_n$-packing with $|P|>\operatorname {vol}(\mathbb {T})$, where $S_n$ is the *n*-dimensional cross-polytope

$$\begin{aligned} S_{n} :=\left\{ (x_1, \ldots , x_n) \in \mathbb {R}^{n} :|x_1| + \cdots + |x_n| \le 2 \ln n \right\} . \end{aligned}$$

Suppose that $\textbf{X} \subseteq \mathbb {T}$ is sampled from the Poisson point process with intensity

$$\begin{aligned} \rho :=\ln |P| - \ln \left( \operatorname {vol}(\mathbb {T})\right) . \end{aligned}$$

Then $\mathbb {P}\left[ \,P \subseteq C_{n} + \textbf{X}\,\right]$ tends to 0 as $n \rightarrow \infty$.

### Proof of Theorem 3.6

Let $\mathbb {T}$, *P*, and $\textbf{X}$ be as in the theorem. Similar to the proof of Theorem 3.5, for every point $\textbf{p} \in P$, let $B_{\textbf{p}}$ be the characteristic function of the event that $\textbf{p} \notin \textbf{X} + C_{n}$.

Since $\operatorname {vol}(C_n) = 1$, the random variable $|\textbf{X} \cap (\textbf{p} - C_n)|$ has the Poisson distribution with mean $\rho \cdot \operatorname {vol}(\textbf{p} - C_n) = \rho$. Therefore, $\mathbb {E}[B_{\textbf{p}}] = \textrm{e}^{-\rho }$. Since $\operatorname {vol}(\mathbb {T})\ge \operatorname {vol}(2C_n)=2^n$, it follows that the sum $B :=\sum _{\textbf{p} \in P}B_{\textbf{p}}$ satisfies

18 $$\begin{aligned} \mathbb {E}[B] = |P|\, \textrm{e}^{-\rho } =\operatorname {vol}(\mathbb {T}) \rightarrow \infty \quad \text {as}\quad n \rightarrow \infty . \end{aligned}$$

Let

$$\begin{aligned} \Sigma _{1}&:=\sum _{\textbf{p} \in P} \mathbb {E}\left[ B_{\textbf{p}} B_{\textbf{p}}\right] = \sum _{\textbf{p} \in P} \mathbb {E}\left[ B_{\textbf{p}}\right] = \mathbb {E}\left[ B\right] \quad \text {and}\quad \\ \Sigma _{2}&:=\sum _{\textbf{p}, \textbf{p}' \in P,~\textbf{p} \ne \textbf{p}'} \left( \mathbb {E}\left[ B_{\textbf{p}} B_{\textbf{p}'}\right] - \mathbb {E}\left[ B_{\textbf{p}}\right] \mathbb {E}\left[ B_{\textbf{p}'}\right] \right) . \end{aligned}$$

### Claim 3.7

We have $$\Sigma _{2} = o\big ( \mathbb {E}\left[ B\right] ^{2} \big )$$.

### Proof of Claim 3.7

Fix two distinct points $\textbf{p}, \textbf{p}' \in P$. Since *P* is an $S_{n}$-packing, we have

$$\begin{aligned} \left( \textbf{p} + S_{n}\right) \cap \left( \textbf{p}' + S_{n}\right) = \emptyset , \end{aligned}$$

which implies that $\textbf{x} = (x_1, \ldots , x_n) :=\textbf{p} - \textbf{p}'$ does not belong to $\textrm{int}(S_{n} - S_{n})$, the interior of $S_{n} - S_{n}$. In particular, $\textbf{x} \not \in \textrm{int}(S_n) \subseteq \textrm{int}(S_n - S_n)$ (since $\textbf{0} \in S_{n}$), and hence,

19 $$\begin{aligned} |x_1| + \cdots + |x_n| \ge 2 \ln n. \end{aligned}$$

Combining it with the AM–GM inequality, we obtain

20 $$\begin{aligned} \operatorname {vol}\left( C_{n} \cap (C_{n} + \textbf{x})\right) = \prod _{i \in [n]} \max \left\{ 1-|x_i|,~0 \right\}&\le \left( \max \left\{ 1-\frac{|x_1|+\cdots +|x_n|}{n},0\right\} \right) ^n \nonumber \\&\le \left( 1- \frac{2\ln n}{n}\right) ^n \le \frac{1}{n^2}. \end{aligned}$$

For sufficiently large $n, B^n_{1/n}$ is contained in $S_n$. Its projection to $\mathbb {T}$ is injective: otherwise two of its points differ by a nonzero $\boldsymbol{\lambda }$ in Λ, and then $\boldsymbol{\lambda }$ lies in $B^n_{2/n}$, which is contained in $4C_n$, contradicting that Λ is a packing lattice of $2C_n$. Since *P* is an $S_n$-packing, the projected translates $\textbf{p}+B^n_{1/n}$ are disjoint. Thus $|P|n^{-n}\nu _n \le \textrm{vol}(\mathbb {T})$, and consequently

21 $$\begin{aligned} \rho ={\ln } |P| - {\ln } \,\textrm{vol}({T}) \le n \ln n - \ln \nu _n = (3/2+o(1))n \ln n = o(n^2).\end{aligned}$$

Similarly to (14), it follows from (20) and (21) that

$$\begin{aligned} \Sigma _{2}&\le \sum _{\textbf{p} \in P} \sum _{\textbf{p}' \in P \setminus \{\textbf{p}\}} \left( \mathbb {E}\left[ B_{\textbf{p}} B_{\textbf{p}'}\right] - \mathbb {E}\left[ B_{\textbf{p}}\right] \mathbb {E}\left[ B_{\textbf{p}'}\right] \right) \\&\le |P|^2 \left( \textrm{exp}\left( -2\rho + \frac{\rho }{n^2} \right) - \textrm{exp}(-2\rho )\right) = \mathbb {E}\left[ B\right] ^{2} \left( \textrm{exp}\left( \frac{\rho }{n^2}\right) - 1\right) \\&= o\big ( \mathbb {E}\left[ B\right] ^{2} \big ), \end{aligned}$$

which proves Claim 3.7. □

It follows from Lemma 3.4 that

$$\begin{aligned}&\mathbb {P}[\,B = 0\,] \le \frac{\textrm{Var}[B]}{\mathbb {E}[B]^{2}}\\&\quad = \frac{1}{\mathbb {E}[B]^{2}} \sum _{\textbf{p}, \textbf{p}' \in P} \left( \mathbb {E}[B_\textbf{p} B_{\textbf{p}'}] - \mathbb {E}[B_\textbf{p}]\, \mathbb {E}[B_{\textbf{p}'}] \right) \\&\quad \le \frac{\Sigma _1 + \Sigma _2}{\mathbb {E}[B]^{2}} = o\left( 1\right) , \end{aligned}$$

which proves that $\mathbb {P}\left[ \,P \subseteq C_{n} + \textbf{X}\,\right]$ tends to 0 as $n \rightarrow \infty$. □

### Proof of Theorem 1.5

By monotonicity, it suffices to prove Theorem 1.5 for some $\rho \ge n\ln n - (1+o(1)) n \ln \ln n$. Now suppose that *P* is a maximal $S_{n}$-packing of $\mathbb {T}$. Since $S_{n}$ is centrally symmetric, it follows that *P* is a $2S_{n}$-covering of $\mathbb {T}$. Consequently,

$$\begin{aligned} |P| \ge \frac{\operatorname {vol}(\mathbb {T})}{\operatorname {vol}(2 S_{n})} = \frac{\operatorname {vol}(\mathbb {T})}{2^n \frac{2^n}{n!} \left( 2 \ln n\right) ^{n}} = (1+o(1))\sqrt{2\pi n} \left( \frac{n}{8 \textrm{e}\ln n}\right) ^n\cdot \operatorname {vol}(\mathbb {T}). \end{aligned}$$

It follows that

$$\begin{aligned} \rho = \ln |P| -\ln \left( \operatorname {vol}(\mathbb {T})\right) \ge n \ln n - (1+o(1)) n \ln \ln n. \end{aligned}$$

By Theorem 3.6,

$$\begin{aligned} \mathbb {P}\left[ \,\mathbb {T} \subseteq \textbf{X} + C_n\,\right] \le \mathbb {P}\left[ \,P \subseteq \textbf{X} + C_n\,\right] = o(1). \end{aligned}$$

This completes the proof of Theorem 1.5. □
